# Standardizing Quality Metrics for Neuraxial Labor Analgesia: A Quality Improvement Framework for Obstetric Anesthesia

**DOI:** 10.7759/cureus.98450

**Published:** 2025-12-04

**Authors:** Shivang S Patel, Nadiya A Persaud, Dean Cirilla, David Vann, Brendan Malik, Mani Vindhya

**Affiliations:** 1 Department of Research, Orlando College of Osteopathic Medicine, Winter Garden, USA; 2 College of Public Health, University of South Florida, Tampa, USA; 3 Department of Anesthesiology, BayCare Medical Group, Tampa, USA

**Keywords:** benchmark, dashboard, obstetric anesthesia, quality improvement research, quality metric

## Abstract

Neuraxial labor analgesia, delivered through epidural or combined spinal-epidural techniques, remains the cornerstone of intrapartum pain management. However, variation in quality measurement continues to limit opportunities for benchmarking and systematic improvement. This study aimed to synthesize existing quality measures and propose a standardized obstetric anesthesia quality improvement framework with clear, measurable benchmarks. A targeted review was conducted using predefined inclusion and exclusion criteria to identify organizational guidelines, peer-reviewed literature, and gray literature sources relevant to neuraxial labor analgesia quality metrics, which were then refined through expert consensus. A multidisciplinary panel consisting of attending anesthesiologists, anesthesiology residents, certified registered nurse anesthetists (CRNAs), and certified anesthesiologist assistants (CAAs) reviewed the synthesized evidence, and all benchmarks were finalized through structured discussion and consensus-based decision-making. The resulting framework outlines proposed operational benchmarks across five domains. Access and timeliness were defined as at least 90% of eligible patients receiving neuraxial analgesia within 30 minutes of request, with a median request-to-placement time of 30 minutes or less. Safety metrics included dural puncture rates below 1%, epidural replacement below 5%, maternal hypotension requiring vasopressors below 10%, and elimination of high neuraxial block or epidural-related infection/hematoma as sentinel events. Effectiveness measures targeted adequate pain relief within 20 minutes in at least 95% of cases and conversion to general anesthesia for cesarean delivery below 1%. Documentation and compliance required complete procedural records, checklist use, and pre- and post-procedure pain scores in at least 95% of cases. Patient experience benchmarks included at least 90% satisfaction with pain management and 95% willingness to choose neuraxial analgesia again. Benchmark thresholds were informed by published evidence, national and international recommendations, findings across included sources, and expert consensus. A consolidated, procedure-specific dashboard can align practice expectations, enable transparent benchmarking, and drive continuous improvement. Adoption of this framework can standardize measurement and promote high-quality, patient-centered obstetric anesthesia care.

## Introduction and background

Neuraxial labor analgesia, administered via standard epidural or combined spinal-epidural techniques, is the most effective method for alleviating the pain of childbirth. It has been utilized for prepartum, parturition, and postpartum for pain management in nearly all obstetrics units across the United States [1]. Given its widespread use in maternal care, the quality and safety of epidural analgesia have become an important focus of national quality initiatives. Maternal mortality and morbidity remain major public health concerns, and modern neuraxial labor analgesia, particularly low-dose local anesthetic techniques combined with opioids, has been shown to provide effective pain relief without increasing labor duration or the rate of operative delivery [2]. Several procedural complications may arise, including inadvertent dural puncture, high neuraxial block, and inadequate pain control, all of which can influence maternal satisfaction and clinical outcomes. There are also several potential side effects, including hypotension, nausea and vomiting, intravascular injection, local anesthetic systemic toxicity, bronchoconstriction, postdural puncture headache, and transient neurological syndrome. Additional risks include nerve injury with possible neuropathy or, rarely, paralysis, as well as epidural hematoma, epidural abscess, meningitis, accidental intrathecal injection with total spinal anesthesia, and osteomyelitis associated with epidural anesthesia [3].

Despite the importance of neuraxial labor anesthesia in obstetric care and its wide range of potential adverse outcomes, quality metrics specific to this procedure remain inconsistently reported across institutions. The American Society of Anesthesiologists (ASA) and the Society for Obstetric Anesthesia and Perinatology (SOAP) have established recommendations for specific aspects of care [4-7]. However, these are often limited and do not holistically provide a standardized framework across all domains necessary for successful procedures. In addition, there are variations in procedural protocols at the institutional level, further limiting the standardization of care. Comparatively, quality frameworks from the Centers for Medicare and Medicaid Services (CMS), the Joint Commission, and the Agency for Healthcare Research and Quality (AHRQ), which emphasize patient safety, timeliness, and documentation, also do not provide a consolidated, obstetric anesthesia-specific dashboard [8-15].

To address these gaps, this paper draws upon the consensus of a multidisciplinary expert panel consisting of attending anesthesiologists (MD and DO), anesthesiology residents, certified registered nurse anesthetists (CRNAs), and certified anesthesiologist assistants (CAAs). Panel members were selected for their clinical expertise, involvement in departmental quality initiatives, and familiarity with neuraxial anesthesia practices. All recommended benchmarks were developed through structured discussion and finalized by consensus.

The paper aims to advance the field of obstetric anesthesia and quality improvement by summarizing neuraxial analgesia-specific quality metrics, aligning them with national benchmarks, and proposing a systemwide framework that can be applied across all obstetric units. By integrating these metrics into defined domains, this paper proposes standardizable benchmarks that can support both clinical excellence and quality improvement initiatives. The following domains are discussed in detail in this paper: access and timeliness, patient safety, effectiveness, documentation and compliance, and patient satisfaction.

## Review

This project was conducted as a quality improvement framework development study. The objective was to synthesize existing guidance and published data to inform a standardized set of quality metrics specific to neuraxial labor analgesia. This was not conducted as a formal systematic review. Rather, a targeted review was performed to synthesize relevant organizational guidelines, peer-reviewed studies, and credible gray literature for the purpose of developing a practical quality improvement framework.

Sources of data

This project was conducted as a quality improvement framework development study. Recommendations and quality measures were collected from leading national organizations, including the ASA, SOAP, CMS, Joint Commission, and AHRQ. To supplement organizational guidelines, peer-reviewed studies were identified through PubMed. Google Scholar and targeted Google searches were used to capture gray literature such as institutional quality dashboards, consensus statements, and practice guidelines not indexed in the major databases.

Search strategy

A targeted review of PubMed was conducted for articles published between January 2000 and December 2024. Searches combined keywords related to neuraxial labor analgesia and quality metrics. The detailed strategies are provided in Table 1.

**Table 1:** Database and web-based search strategies for neuraxial labor analgesia quality metrics This table summarizes the structured search strategies applied across PubMed, Google Scholar, and supplementary web-based searches. Keywords and controlled vocabulary terms were combined to capture studies related to neuraxial labor analgesia, obstetric care, and quality metrics or improvement initiatives.

Source	Search strategy
PubMed	“neuraxial analgesia” OR “epidural analgesia” OR “combined spinal epidural” AND “labor” OR “childbirth” OR “obstetric” AND “quality metrics” OR “benchmark*” OR “performance measure*” OR “quality improvement”
Google Scholar	“neuraxial labor analgesia” AND (“quality metrics” OR “benchmark*” OR “quality improvement”) filter: 2000–2024
Google (gray literature)	“neuraxial labor analgesia” AND “quality improvement” site:.org OR site:.gov (used to identify society guidelines, institutional dashboards, and national reports)

Inclusion and exclusion criteria

Articles were included if they were peer-reviewed, published in English between 2000 and 2024, and focused on neuraxial labor analgesia, including epidural, spinal, or combined spinal-epidural techniques. Eligible studies were required to report on quality metrics, performance benchmarks, or quality improvement initiatives, and guidelines, consensus statements, or quality frameworks issued by national or international organizations were also considered. Excluded from review were case reports, editorials, and conference abstracts without full text, as well as studies limited to pharmacologic or technical aspects of anesthesia that did not address quality metrics. In addition, research involving non-obstetric populations, such as postoperative or chronic pain management, and articles not published in English were excluded.

Study selection

The database searches and organizational guideline reviews yielded a broad set of publications relevant to neuraxial labor analgesia. Titles and abstracts were screened for relevance to quality metrics and benchmarking in obstetric anesthesia. Articles that did not address quality outcomes, were focused solely on pharmacologic or technical aspects, or were non-obstetric in nature were excluded. The final body of evidence included a combination of peer-reviewed studies, consensus statements, and national quality frameworks, which were synthesized to inform the proposed benchmarks.

Framework development

Five domains were identified as central to obstetric anesthesia quality: access and timeliness, safety, effectiveness, documentation and compliance, and patient satisfaction. Within each domain, specific metrics (i.e., median time from request to placement, dural puncture rate, adequacy of pain relief, completeness of documentation, and patient satisfaction scores) were defined based on convergent findings across included studies and organizational guidelines. Additional benchmarks were informed by best-practice recommendations and internal quality initiatives.

**Figure 1:**
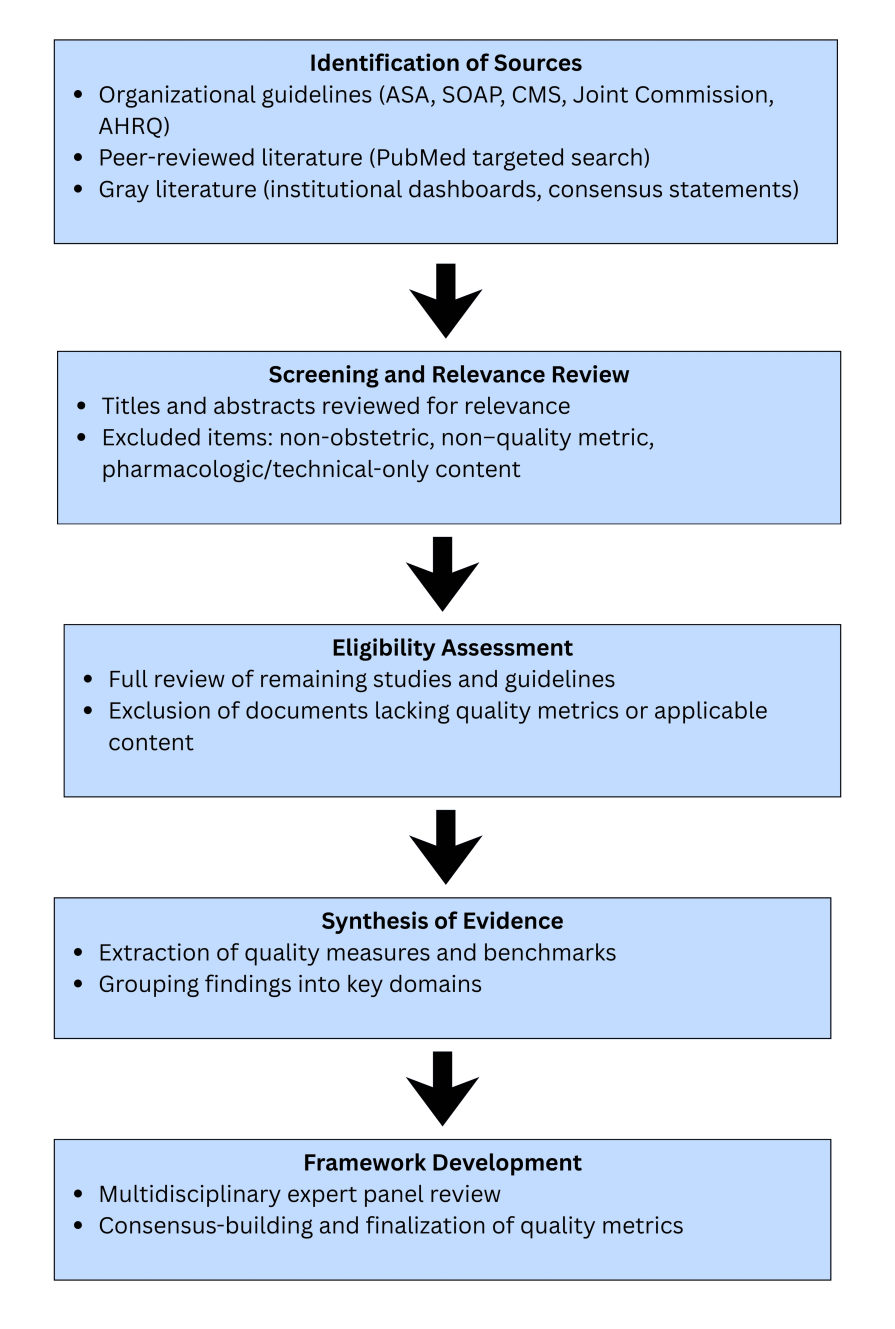
Methodological workflow for framework creation ASA: American Society of Anesthesiologists; SOAP: Society for Obstetric Anesthesia and Perinatology; CMS: Centers for Medicare and Medicaid Services; AHRQ: Agency for Healthcare Research and Quality Infographic designed by Nadiya A. Persaud on Canva (Canva Inc., Perth, Australia)

A multidisciplinary team of clinicians, obstetric anesthesiologists, obstetricians, and quality improvement specialists reviewed the synthesized evidence to establish operational definitions and finalize benchmarks. The methodological framework used to develop the benchmarks and metrics is illustrated in Figure 1.

Key domains

Obstetrics anesthesia quality measures were evaluated across four key domains: access and timeliness, safety, effectiveness, and documentation and patient satisfaction.

In the access and timeliness domain (Table 2), these benchmarks focus on prompt epidural access for eligible patients for ≥90% within ≤30 minutes from a request-to-placement time. The administration rate is ≥90% within 30 minutes. These recommendations were based on multiple national guidelines and some internal targets from academic centers.

**Table 2:** Access and timeliness Proposed benchmarks for access and timeliness based on the cited sources. SOAP: Society for Obstetric Anesthesia and Perinatology

Domain	Quality metrics	Benchmark	Source
Access and timeliness	Epidural access rate for eligible laboring patients	≥90%	SOAP recommendations and internal academic center targets [6]
	Median time from request to placement	≤30 minutes	SOAP statements and Leapfrog quality frameworks [6,16]
	Administration within 30 minutes of request	≥90%	Mayo Clinic and Leapfrog quality frameworks [16,17]

In the safety domain (Table 3), complications were closely monitored with targets of <1% dural puncture rate, <5% replacement rate, and zero tolerance for high spinal/high block. Maternal hypotension requiring vasopressors was expected in <10% of cases, and epidural-related infections or hematomas were expected to remain rare based on national organizations and recent studies. 

**Table 3:** Safety Proposed benchmarks for safety based on cited sources SOAP: Society for Obstetric Anesthesia and Perinatology; ASA: American Society of Anesthesiologists

Domain	Quality metrics	Benchmark	Source
Safety	Dural puncture rate (wet tap)	<1%	ASA Closed Claims data and expert consensus [17,18]
	Epidural replacement rate	<5%	Internal institutional targets and trends reported in SOAP surveys [6,7]
	High spinal/high block	0%	Zero tolerance safety goal derived from national patient safety principles and expert consensus
	Maternal hypotension requiring vasopressors	<10%	SOAP guidelines and observational data [6,7]
	Epidural-associated infection or hematoma	0%	Zero tolerance safety expectation based on rarity reported in national datasets and expert consensus

In the effectiveness domain (Table 4), the benchmark was set to ≥95% for adequate pain relief within 20 minutes. The conversion to general anesthesia for cesarean delivery was expected to remain <1% based on SOAP and ASA recommendations.

**Table 4:** Effectiveness Proposed benchmarks for effectiveness based on cited sources. SOAP: Society for Obstetric Anesthesia and Perinatology; ASA: American Society of Anesthesiologists

Domain	Quality metrics	Benchmark	Source
Effectiveness	Adequate pain relief within 20 minutes	≥95%	SOAP guidelines and expert consensus [6,7]
	Conversion to general anesthesia for cesarean section	<1%	ASA and SOAP quality measures and supported by expert consensus [4-7]

In the documentation and patient satisfaction domain (Table 5), documentation goals encompass 100% for completeness of records and timeout checklist use. Additionally, compliance with pain score documentation should be ≥95%. Patient satisfaction benchmarks targeted ≥90% satisfaction with pain management and ≥95% willingness to choose epidural in the future, which was based on the Hospital Consumer Assessment of Healthcare Providers and Systems, internal quality improvement, and local surveys. 

**Table 5:** Documentation and compliance and patient satisfaction Proposed benchmarks for documentation and compliance and patient satisfaction based on cited sources. CMS: Centers for Medicare and Medicaid Services; AHRQ: Agency for Healthcare Research and Quality; HCAHPS: Hospital Consumer Assessment of Healthcare Providers and Systems

Domain	Quality metrics	Benchmark	Source
Documentation and compliance	Complete documentation of the procedure	100%	CMS and Joint Commission documentation requirements; proposed as a 100% institutional target [8-10]
	Timeout checklist usage	100%	National Patient Safety Goals; proposed as a 100% institutional target [12]
	Pre- and post-pain score documentation	≥95%	AHRQ best-practice recommendations and expert consensus [13]
Patient satisfaction	Satisfaction with pain management	≥90%	HCAHPS patient experience standards and internal Press Ganey data [19,20]
	Willingness to choose an epidural in the future	≥95%	Local patient experience surveys and internal quality improvement data

These benchmarks provide a comprehensive framework for assessing performance and guiding quality improvement initiatives in obstetric anesthesia.

Access and timeliness

Accessibility and punctuality of neuraxial analgesics during parturition are very critical quality measures. Delays are associated with increased pain and suffering, with a poor maternal satisfaction rate. In women with a medical indication for epidural analgesia, including high-risk patients such as those experiencing preterm labor, timely administration of neuraxial analgesia is associated with a significant reduction in severe maternal morbidity [20,21].** **Acknowledging national recommendations from the SOAP, Mayo Clinic, and Leapfrog, along with internal targets for academic centers, accessibility benchmark should be greater than 90% of all women in childbirth within less than 30 minutes at the time of request (Table 2). Monitoring these quality measures ensures patients receive effective and compassionate pain relief in a timely manner and potentially reduces the risk of mortality.

Safety

Patient safety should remain the integral measure of obstetric anesthesia. Complications like inadvertent dural puncture, epidural-associated infection, hematoma, and high spinal block are rare but severe, which warrant continuous monitoring [22]. For many of these complications, the benchmark should be zero tolerance (Table 3). The ASA Closed Claims database and certain studies report a benchmark of less than 1% for dural puncture incidence. Similarly, the replacement rate of epidurals should remain below 5% because higher rates suggest issues with catheter placement or management. While several complications warrant a zero tolerance benchmark as outlined in Table 3, the literature shows that epidural catheter replacement rates vary across techniques, patient populations, and institutional practices. Reports from large obstetric cohorts describe replacement or failure rates in the range of 3-6% for the general population [23], with higher rates in specific groups such as super-obese parturients, where failure rates of approximately 14% have been observed [24]. Older data evaluating epidural top-ups for cesarean delivery also show higher inadequacy rates in certain settings [25]. Due to this variation, a single rigid threshold may not reflect the realities of all clinical environments. Institutions should therefore establish evidence-informed benchmarks and monitor their own catheter replacement patterns over time to identify meaningful quality deviations. Another measure of maternal hypotension requiring vasopressors is a common complication for which SOAP and observational cohort studies support a benchmark of below 10%. These quality measures ensure patients' safety against common adverse outcomes.

Effectiveness

The effectiveness of the neuraxial analgesics is the next important matrix after procedural safety. It is central to patients' satisfaction and clinical outcomes. Timely and effective pain relief ensures that laboring patients do not experience prolonged suffering, which can negatively impact both the physical and emotional course of labor [26]. Adequate pain relief within 20 minutes of administration is recommended in more than 90% of the laboring patients (Table 4). Another critical outcome is the conversion to general anesthesia for cesarean delivery, with a recommended benchmark of less than 1%. While conversion may occasionally be necessary when neuraxial analgesia is suboptimal, recommended benchmarks emphasize that conversion during elective or scheduled cesarean deliveries should remain exceedingly rare. Higher rates may be observed in emergency or intrapartum cases due to clinical urgency and the need for rapid anesthesia initiation.

Documentation and compliance and patient satisfaction

As per regulatory bodies, including the CMS and Joint Commission, complete documentation and adherence to safety checklists are essential to sustain accreditation [8-10]. Institutions should strive for a 100% completion rate of procedural notes and use of time-out checklists, and greater than 95% compliance with pre- and post-procedural pain score documentation (Table 5) is essential for both patient safety and institutional accountability. These practices also enable quality improvement initiatives by ensuring reliable data collection. Additionally, patient experiences are a critical factor in determining the success of the procedure. Satisfaction with pain management and willingness to choose an epidural analgesic in the future provide a direct measure of the patients' perception of safety, effectiveness, and respect during care. Benchmarks of ≥90% satisfaction and ≥95% willingness to repeat the procedure are consistent with national patient experience surveys, including HCAHPS and Press Ganey data (Table 5). This also underscores the importance of a patient-centered approach in obstetric anesthesia.

## Conclusions

Obstetric anesthesia quality metrics provide a structured way to evaluate the safety, effectiveness, and patient satisfaction of neuraxial analgesics. Adopting systemwide metrics creates a standardized dashboard that reinforces quality improvement and accountability among healthcare institutions. The suggested framework, based on the recommendations of national organizations, not only improves maternal safety and satisfaction but also assists in assessing benchmarking across institutions. This would foster a culture of transparency and continuous improvement, which is essential for modern healthcare to provide optimal patient-centered care and improve the overall safety and experience of childbirth.
